# Comparing associations of handgrip strength and chair stand performance with all-cause mortality—implications for defining probable sarcopenia: the Tromsø Study 2015–2020

**DOI:** 10.1186/s12916-023-03172-3

**Published:** 2023-11-20

**Authors:** Jonas Johansson, Sameline Grimsgaard, Bjørn Heine Strand, Avan A. Sayer, Rachel Cooper

**Affiliations:** 1 Department of Community Medicine, UiT The Arctic University of Norway, Tromsø, Norway; 2 Norwegian National Centre for Ageing and Health, Vestfold Hospital Trust, Tønsberg, Norway; 3 Department of Geriatric Medicine, Oslo University Hospital, Oslo, Norway; 4 Department for Physical Health and Ageing, Norwegian Institute of Public Health, Oslo, Norway; 5 AGE Research Group, Translational and Clinical Research Institute, Faculty of Medical Sciences, Newcastle University, Newcastle Upon Tyne, UK; 6 NIHR Newcastle Biomedical Research Centre, Newcastle University, Newcastle Upon Tyne NHS Foundation Trust and Cumbria, Northumberland, Tyne and Wear NHS Foundation Trust, Newcastle Upon Tyne, UK

**Keywords:** Handgrip strength, Chair stand performance, All-cause mortality, Sarcopenia

## Abstract

**Background:**

Widely adopted criteria suggest using either low handgrip strength or poor chair stand performance to identify probable sarcopenia. However, there are limited direct comparisons of these measures in relation to important clinical endpoints. We aimed to compare associations between these two measures of probable sarcopenia and all-cause mortality.

**Methods:**

Analyses included 7838 community-dwelling participants (55% women) aged 40–84 years from the seventh survey of the Tromsø Study (2015–2016), with handgrip strength assessed using a Jamar + Digital Dynamometer and a five-repetition chair stand test (5-CST) also undertaken. We generated sex-specific *T*-scores and categorised these as “not low”, “low”, and “very low” handgrip strength or 5-CST performance. Cox Proportional Hazard regression models were used to investigate associations between these two categorised performance scores and time to death (up to November 2020 ascertained from the Norwegian Cause of Death registry), adjusted for potential confounders including lifestyle factors and specific diseases.

**Results:**

A total of 233 deaths occurred (median follow-up 4.7 years) with 1- and 5-year mortality rates at 3.1 (95% confidence interval [CI] 2.1, 4.6) and 6.3 (95% CI 5.5, 7.2) per 1000 person-years, respectively. There was poor agreement between the handgrip strength and 5-CST categories for men (Cohen’s kappa [*κ*] = 0.19) or women (*κ* = 0.20). Fully adjusted models including handgrip strength and 5-CST performance mutually adjusted for each other, showed higher mortality rates among participants with low (hazard ratio [HR] 1.22, 95% CI 0.87, 1.71) and very low (HR 1.68, 95% CI 1.02, 2.75) handgrip strength compared with the not low category. Similar associations, although stronger, were seen for low (HR 1.88, 95% CI 1.38, 2.56) and very low (HR 2.64, 95% CI 1.73, 4.03) 5-CST performance compared with the not low category.

**Conclusions:**

We found poor agreement between *T*-score categories for handgrip strength and 5-CST performance and independent associations with mortality. Our findings suggest that these tests identify different people at risk when case-finding probable sarcopenia. As discussions on an international consensus for sarcopenia definitions proceed, testing both handgrip strength and chair stand performance should be recommended rather than viewing these as interchangeable assessments.

**Supplementary Information:**

The online version contains supplementary material available at 10.1186/s12916-023-03172-3.

## Background

Maintaining muscle strength and physical function in later life is now recognised as an important element of healthy ageing based on evidence that lower levels of muscle function are associated with a range of adverse outcomes including premature mortality, mobility disability, fractures, cognitive decline, and hospitalisations [1–4]. Muscle strength is known to gradually decrease after midlife, and given the globally ageing population, increased preservation of this physical capacity will likely contribute to healthy ageing and reduced future burden on healthcare systems [5]. The compelling evidence showing strong associations between markers of low muscle strength and important health outcomes led the European Working Group on Sarcopenia in Older People to revise their definition (EWGSOP2) to include low muscle strength as the primary step in sarcopenia diagnosis [6].

The EWGSOP2 definition (also supported by Australia and New Zealand) recommends using handgrip strength or the five-repetition chair stand test (5-CST) to identify low muscle strength and establish the presence of probable sarcopenia [6, 7]. This implies that handgrip strength and chair stand performance can be used interchangeably. Handgrip strength, as a direct measure of muscle strength, is the more widely employed test in clinics and research studies, likely due to being easier to standardise, having an earlier established protocol, and holding higher practicality in very old individuals and inpatients [8–12]. In comparison, while leg muscle strength explains a considerable part of 5-CST performance, this test also relies on a variety of psychological, balance, and sensorimotor factors [13]. Additionally, assessing 5-CST performance is more demanding than testing handgrip strength, as indicated by the higher proportion of individuals unable to complete the test at older ages [12].

In studies that have assessed both handgrip strength and chair stand performance, there is evidence of only modest correlation and low levels of agreement between classifications of probable sarcopenia defined using the two measures [14, 15]. However, few studies have conducted direct comparisons of the associations between these two measures and important clinical endpoints [16], as required to fully understand the implications of these differences for case finding probable sarcopenia [6]. The current study aimed to investigate the agreement between handgrip strength and 5-CST performance and whether these assessments were comparatively associated with all-cause mortality in a population-based sample of community-dwelling adults aged 40–84 years.

## Methods

### Study sample description

The study sample was drawn from the seventh survey of the population-based Tromsø Study (Tromsø7), which took place during 2015–2016 in Tromsø, Norway. The data collection procedures have been described in detail elsewhere [17]. In summary, all inhabitants in Tromsø municipality aged 40 years and older (*n* = 32,591) were invited to participate, and 21,083 (65%) of these attended basic examinations. Of those that attended, a randomised sub-sample of 9253 participants were also invited to extended examinations including clinical tests of physical performance. Here, 7838 (85%) community-dwelling men and women aged 40–84 completed assessments of muscle strength and physical function and constitute the final sample for the current study.

### Handgrip strength and 5-CST data collection

Handgrip strength was assessed according to procedures outlined in the Southampton protocol [11]. Participants were seated and instructed to hold a Jamar + Digital Dynamometer (Patterson Medical, Warrenville, IL, USA) with a 90° elbow angle. They were asked to squeeze the dynamometer with maximal effort during six attempts (three in each hand) and the highest value from all attempts was used in the present study.

Lower extremity physical function was assessed using the 5-CST, part of the Short Physical Performance Battery [18]. Participants were instructed to perform five complete raises from a chair as fast as possible with their arms folded across the chest. A test instructor used a stopwatch to record the time elapsed between initiation of the first rise and until the participant stood up again after the fifth repetition. Each participant practiced one rise before the actual test. 5-CST non-completion was recorded if the participant took more than 60 s, used their hands for support, or was unable to perform the test due to safety issues.

### All-cause mortality

The study outcome was date of death from all causes obtained from the Norwegian Cause of Death Registry. For the purposes of analyses, participants were considered to have entered the study on the date of their individual examination during Tromsø7 in 2015–2016 and were censored on their date of death, the date they were lost to follow-up due to migration, or at the end of follow-up (3 November 2020), whichever occurred first.

### Potential confounders

Potential confounders were chosen a priori based on evidence of their associations with muscle strength, physical function, and mortality in existing literature [16, 19, 20]. All variables listed were ascertained during the same assessment as the handgrip strength and 5-CST measurements in Tromso7 during 2015–2016. Height (m) and weight (kg) were measured with participants in light clothing without shoes, and body mass index (BMI) was calculated as kg/m^2^. Participants self-reported smoking status (currently smoking yes, no), education level (primary school, upper secondary school, college or university < 4 years, college or university ≥ 4 years), and current disease status (cardiovascular disease; rheumatoid arthritis; respiratory disease). In addition, they reported leisure time physical activity according to the Saltin-Grimby Physical Activity Level Scale (inactive, lightly physical active, moderately physically active, or vigorously physically active) [21].

### Statistical analysis

Descriptive data are presented using means and standard deviations (SD), or by number of participants (*n*) and percentages (%). To standardise the comparison of associations of HGS and 5CST with mortality, we decided to take advantage of the broad age range of our sample and calculate sex-specific *T*-scores for handgrip strength and the 5-CST, defined as the number of standard deviations above or below the mean value in the youngest age group (40–44 years). The EWGSOP2 consensus statement has recommended using regional normative populations when available, and our *T*-score cutoffs presented below are similar to the ones used in osteoporosis and sarcopenia definitions [6, 22]. We thus used the *T*-scores to classify participants as having “not low” (*T*-score >  − 1), “low” (− 1 to − 2.49), or “very low” (≤ − 2.5) handgrip strength, and similarly “not low” (< 1), “low” (1 to 2.49), or “very low” (≥ 2.5) 5-CST performance. 5-CST non-completers were added to the “very low” category because exclusion of such participants may introduce bias [16]. We used Cohen’s Kappa (*κ*) to evaluate the agreement between the handgrip strength and 5-CST *T*-score categories, and a linear-by-linear test for trend to compare differences between the two performance measures by age group and sex.

Associations between both performance-based measures (modelled in three categories) and all-cause mortality were examined using Cox proportional hazard regression models, using age as the timescale [23]. Model 1 was adjusted for sex; we formally tested interactions between sex and handgrip strength and 5-CST in this model, and where there was no evidence of this, subsequent models were adjusted for sex. Model 2 was additionally adjusted for height, BMI, physical activity, education level, smoking status, cardiovascular disease, rheumatoid arthritis, and respiratory disease. Model 3 (final model) included additional adjustment for the other performance-based measure. We performed visual inspection of plots and tested the Schoenfeld residuals; no severe violation of the proportional hazard assumption was found. We also ran an additional adjusted Cox model to further evaluate the relationship between handgrip strength and 5-CST performance, by allocating all participants to one of nine different categories where their handgrip strength and 5-CST status was combined (coded very low—very low to not low—not low). This variable was then examined in association with all-cause mortality.

To take account of missing data on covariates (which ranged 0.2 to 6%; Table 1) in our survival models, these data were assumed to be missing at random and multiple imputation was undertaken with the inclusion of the Nelson-Aalen estimator of cumulative (baseline) hazard, *H(T*). Our main analyses are run across 30 imputed datasets with results combined using Rubin’s rules [24, 25]. For comparison purposes, we ran a complete case analysis, and these findings are presented as additional data (Additional file 1: Table S1).

**Table 1:** Participant characteristics assessed in 2015–2016 (maximum *N* = 7838^a^). The Tromsø Study 2015–2016

Parameter	Mean (SD) or *n* (%)	Observations (*n*)	% Missing
Age (years)	63.15 (10.47)	7838	0.00
Sex (female)	4274 (54.53)	7838	0.00
Height (m)		7823	0.19
Women	1.63 (0.06)		
Men	1.77 (0.07)		
Weight (kg)		7821	0.22
Women	71.93 (13.21)		
Men	87.02 (13.68)		
BMI (kg/m^2^)		7821	0.22
Women	26.93 (4.79)		
Men	27.78 (3.91)		
Handgrip strength (kg)		7838	0.00
Women	28.10 (5.61)		
Men	47.80 (9.35)		
5-CST time (s)		7838	0.00
Women	9.96 (3.26)		
Men	9.11 (2.88)		
Current smoker (yes)	963 (12.41)	7759	1.01
Education level		7656	2.32
Primary	2254 (29.44)		
Upper secondary	2133 (27.86)		
College/university < 4 years	1414 (18.47)		
College/university ≥ 4 years	1855 (24.23)		
Leisure time physical activity level		7494	4.39
Inactive	975 (13.01)		
Lightly physical active	4602 (61.41)		
Moderately physical active	1759 (23.47)		
Vigorously physical active	158 (2.11)		
CVD (yes)	623 (8.17)	7626	2.70
Rheumatoid arthritis (yes)	391 (5.29)	7392	5.69
Respiratory disease (yes)	325 (4.32)	7519	4.07

*BMI*, body mass index; *5-CST*, 5-repetition chair stand test; *CVD*, cardiovascular disease

Numbers are mean (SD) for continuous parameters and *n* (%) for categorical parameters

^a^Sample with complete data on handgrip strength and 5-CST performance

We performed two sensitivity analyses. First, to assess potential reverse causation bias driven by pre-existing disease (above and beyond adjustments made for health status in our main models), by excluding deaths occurring during the first 2 years of follow-up (*n* = 66) and re-running the main three models. Second, to enable comparison of our study findings with those from studies that have applied the cutoffs recommended in the EWGSOP2 guidelines, we reran our main analyses with handgrip strength and 5-CST categorised using the EWGSOP2 cut-points [6]. All analyses were performed using STATA version 17.0 (StataCorp, College Station, TX, USA).

## Results

Of 7838 participants, 233 died (56 from cardiovascular disease, 116 from cancer, 61 from other causes) over a median follow-up time of 4.7 years (interquartile range 4.4–5.1). This represented a 1-year mortality rate of 3.1 (95% confidence interval [CI] 2.1, 4.6) per 1000 person-years and a 5-year mortality rate of 6.3 (95% CI 5.5, 7.2) per 1000 person-years. As shown in Table 1, mean (SD) age was 63.2 years (10.5), 54.5% of the sample were women and the mean (SD) BMI in women and men was 26.9 (4.8) and 27.8 kg/m^2^ (3.9), respectively (Table 1).

Handgrip strength and 5-CST times are presented for each age group in Table 2. In both women and men, mean handgrip strength and chair rise performance were lower at older ages (*P* for trend < 0.001 for all). Participants unable to complete the 5-CST were prevalent in all age groups for women (*n* = 66), although this increased from age 70 years. There were fewer non-completers among men (*n* = 31) and a less prominent increase in older age groups compared to that observed for women (Table 2).

**Table 2:** Handgrip strength and 5-CST time stratified by age group and sex (*N* = 7838). The Tromsø Study 2015–2016

Age group (years)	*N*	Handgrip strength (kg)	5-CST time (s)	5-CST non-completer, *n* (%)
Women				
40–44	281	33.38 (5.68)	7.83 (2.13)	1 (0.36)
45–49	316	32.60 (5.09)	8.17 (2.44)	1 (0.32)
50–54	328	30.69 (5.50)	8.66 (2.52)	3 (0.92)
55–59	428	29.75 (4.98)	8.82 (2.29)	2 (0.47)
60–64	832	28.79 (4.42)	9.43 (2.68)	5 (0.60)
65–69	855	27.18 (4.67)	10.47 (3.19)	7 (0.82)
70–74	660	25.63 (4.78)	11.40 (3.62)	14 (2.12)
75–79	390	23.98 (4.63)	11.78 (3.13)	18 (4.62)
80 +	184	22.56 (4.54)	12.68 (4.24)	15 (8.15)
*P*-value for trend		< 0.001	< 0.001	
Men				
40–44	231	55.67 (8.40)	7.49 (2.05)	0 (0.00)
45–49	263	56.41 (8.11)	7.95 (1.93)	2 (0.76)
50–54	249	53.24 (8.68)	8.05 (2.42)	2 (0.80)
55–59	318	51.76 (8.29)	8.41 (2.22)	1 (0.31)
60–64	743	49.11 (7.88)	8.75 (2.45)	4 (0.54)
65–69	744	46.11 (7.44)	9.19 (2.79)	5 (0.67)
70–74	525	43.47 (7.63)	9.79 (2.69)	5 (0.95)
75–79	328	40.56 (7.08)	10.72 (3.07)	5 (1.52)
80 +	163	36.99 (6.84)	12.19 (4.63)	7 (4.29)
*P*-value for trend		< 0.001	< 0.001	

*5-CST*, 5-repetition chair stand test

Numbers for handgrip strength and 5-CST are mean (SD)

Table 3 shows the resulting sex-specific handgrip strength and 5-CST cutoffs from generating *T*-scores. Approximately 40% of participants were classified as having low handgrip strength and 5% as having very low handgrip strength. For 5-CST performance, roughly 23 and 5% of all participants had low or very low 5-CST performance, respectively. There was a higher representation of women in the low or very low categories of handgrip strength and 5-CST performance compared with men. The level of agreement between the *T*-score categories of handgrip strength and 5-CST was very low for both women (*κ* = 0.20, 95% CI 0.17, 0.22) and men (*κ* = 0.19, 95% CI 0.16, 0.21) (Table 3).

**Table 3:** *T*-score categories and cutoffs for handgrip strength and 5-CST time (*N* = 7838). The Tromsø Study 2015–2016

Variable	Women (*n* = 4274)				Men (*n* = 3564)			
	**Cutoff**	***N***	**%**	***κ* (95% CI)**	**Cutoff**	***N***	**%**	***κ* (95% CI)**
Grip strength (kg)								
*T*-score > − 1 (not low)	> 27.7	2205	51.6		> 46.3	1986	55.7	
*T*-score − 1 to − 2.49 (low)	27.7–19.4	1837	43.0		46.3–32.4	1432	40.2	
*T*-score ≤ − 2.5 (very low)	≤ 19.3	232	5.4		≤ 32.3	146	4.1	
				0.20 (0.17, 0.22)^a^				0.19 (0.16, 0.21)^b^
5-CST performance (s)								
*T*-score < 1 (not low)	< 11.1	2977	69.6		< 10.4	2615	73.3	
*T*-score 1 to 2.49 (low)	11.1–15.9	1063	24.9		10.4–14.6	779	21.9	
*T*-score ≥ 2.5 (very low)	≥ 16.0	234	5.5		≥ 14.7	170	4.8	

*5-CST*, 5-repetition chair stand test

^a^Agreement (Cohen’s kappa) between handgrip strength and 5-CST categories for women

^b^Agreement (Cohen’s kappa) between handgrip strength and 5-CST categories for men

The *T*-scores are based on the mean value of the youngest age group (40–44 years) for women and men separately

Results from Cox proportional hazard regression models are shown in Table 4. In model 1, adjusted only for age (as timescale) and sex, there was a tendency for higher mortality with lower handgrip strength as seen in participants in the low (hazard ratio [HR] 1.30, 95% CI 0.94, 1.79) and very low (HR 2.23, 95% CI 1.43, 3.47) categories compared with participants in the not low category. Adjustment for additional confounders had no impact on the estimates but adjustment for 5-CST status in model 3 attenuated the scale of the HR for mortality in participants with low handgrip strength (HR 1.22, 95% CI 0.87, 1.71), and to a larger extent in those with very low handgrip strength (HR 1.68, 95% CI 1.02, 2.75). Corresponding estimates for 5-CST performance in model 1 also revealed a trend of higher mortality with lower performance, as indicated when comparing low (HR 2.09, 95% CI 1.55, 2.81) and very low (HR 3.51, 95% CI 2.41, 5.11) categories with not low. The scale of the HR for low 5-CST performance was attenuated in model 3 (HR 1.88, 95% CI 1.38, 2.56), which included adjustment for handgrip strength. This attenuation was larger for the very low category (HR 2.64, 95% CI 1.73, 4.03) (Table 4).

**Table 4:** Cox proportional hazard regression models for all-cause mortality (*N* = 7838). The Tromsø Study 2015–2020

Variable	*N*	Deaths	MR^a^	Model 1^b^		Model 2^c^		Model 3^c^	
				**HR**	**95% CI**	**HR**	**95% CI**	**HR**	**95% CI**
Handgrip strength									
Not low	4191	62	3.13	1.00	Ref	1.00	Ref	1.00	Ref
Low	3269	132	8.64	1.30	0.94, 1.79	1.36	0.98, 1.91	1.22	0.87, 1.71
Very low	378	39	22.87	2.23	1.43, 3.47	2.29	1.43, 3.68	1.68	1.02, 2.75
5-CST performance									
Not low	5592	88	3.35	1.00	Ref	1.00	Ref	1.00	Ref
Low	1842	97	11.22	2.09	1.55, 2.81	1.94	1.43, 2.64	1.88	1.38, 2.56
Very low	404	48	26.44	3.51	2.41, 5.11	3.01	2.02, 4.50	2.64	1.73, 4.03

*MR*, mortality rate; *HR*, hazard ratio; *CI*, confidence interval; *5-CST*, 5-repetition chair stand test

^a^Mortality rate per 1000 person-years

^b^Tests of sex interaction: low handgrip strength (*p* = 0.987), very low handgrip strength (*p* = 0.156), low 5-CST performance (*p* = 0.099), very low 5-CST performance (*p* = 0.654)

^c^Estimates are from models run across 30 imputed datasets combined using Rubin’s rules

Model 1: adjusted for age (as timescale) and sex

Model 2: adjusted for model 1 + height, BMI, leisure time physical activity, education, smoking status, cardiovascular disease, rheumatoid arthritis, respiratory disease

Model 3: adjusted for model 1 and model 2 + handgrip strength status or 5-CST status

**Fig. 1:**
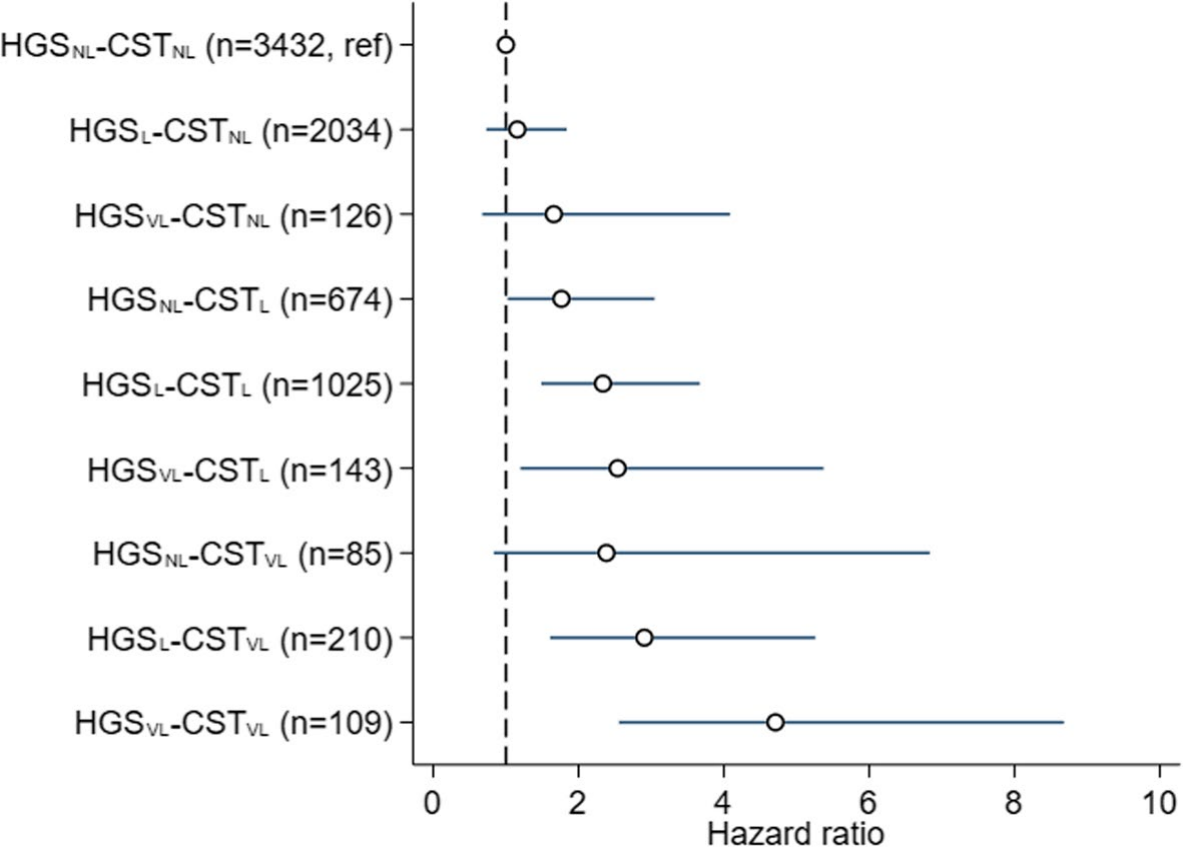
Cox proportional hazard regression showing how different combinations of handgrip strength and 5-CST were associated with mortality. Model used age as timescale and were adjusted for sex, height, BMI, physical activity, education level, smoking status, cardiovascular disease, rheumatoid arthritis and respiratory disease. Estimates are from a model run across 30 imputed datasets combined using Rubin’s rules. HGS, handgrip strength; CST, chair stand test; NL, not low; L, low; VL, very low. The Tromsø Study 2015–2020

Figure 1 illustrates the distribution of different combinations of handgrip strength and 5-CST statuses and their joint associations with mortality. Over a third of participants (*n* = 2917, 39%) had a performance level in one measure that was discordant with the other (e.g. low handgrip strength but very low or not low 5-CST performance). A pattern of higher mortality across the combinations was observed, with the highest mortality rates found in the group (*n* = 109) classified as having very low handgrip strength and very low 5-CST performance (HR 4.71, 95% CI 2.56, 8.68) (Fig. 1).

The first sensitivity analysis showed that when early deaths (*n* = 66) within the first 2 years of follow-up were excluded from analyses, associations between handgrip strength and all-cause mortality became weaker whereas those with 5-CST performance remained (Additional file 1: Table S2). The second sensitivity analysis showed stronger associations between handgrip strength and mortality (HR 3.17, 95% CI 1.91, 5.28) than between 5-CST and mortality (HR 1.85, 95% CI 1.31, 2.63) in fully adjusted models when EWGSOP2 cutoffs were applied (Additional file 1: Table S3).

## Discussion

In this study of Norwegian men and women aged 40 to 84 years, we found limited agreement between *T*-score categories of handgrip strength and 5-CST, suggesting that poor performance in one of the tests does not necessarily identify groups of individuals with poor performance in the other test. Despite this high level of discordance, we found higher all-cause mortality rates over ~ 5 years of follow-up in participants with lower levels of either handgrip strength or 5-CST performance. There were indications from mutually adjusted models that these relationships were independent and that overall associations for 5-CST status were stronger than those for handgrip strength. We also identified different groups of individuals with high mortality rates when assessing the combined effects of the two tests, suggesting that use of both performance measures may be important for evaluating health status, especially as the highest HR was observed in participants with concurrent very low handgrip strength and 5-CST performance.

To our knowledge there have been very few direct and standardised comparisons of the associations of handgrip strength and 5-CST performance with all-cause mortality. In an analysis of a British birth cohort study that followed 2766 men and women between ages 53 and 66, all-cause mortality rates were higher in participants with lower levels of handgrip strength and chair rise time, although the association was stronger for handgrip strength [16]. These findings differ from the current study where instead the 5-CST showed a stronger association with mortality than handgrip strength. Disparities could potentially be explained by differences in study sample ages and the variation in chair stand test protocols used, as the 5-CST is less likely to reflect endurance capacity compared with the 10-repetition CST used in the British birth cohort study [26]. Recent findings from the Toledo Study for Healthy Ageing, investigating 1928 participants over 7.5 years, are partially similar to our study, as they reported higher mortality in participants with very low 5-CST-derived muscle power independent of handgrip strength [27]. We extend these findings through our two-way comparison by also showing how handgrip strength estimates are affected by 5-CST adjustment. Of note, recent findings from the RESORT study on 1250 older patients showed that handgrip strength but not 5-CST performance was associated with re-hospitalisation and 1-year mortality [28]. Their findings contrast with ours and are likely explained by the differences in age and hence overall mortality rate, demographics, setting, and follow-up time, as we included a younger sample of community-dwelling participants followed over ~ 5 years. It should be noted here that our sensitivity analyses excluding deaths within the first 2 years severely weakened the association between handgrip strength and mortality. In addition, there were considerable differences in how many RESORT participants were unable to complete the two tests (handgrip strength: 7.6%, 5-CST: 76.8%), which affects comparability with our study where there were relatively few 5-CST non-completers (1.2%). Nonetheless, their conclusion is similar to ours in that the two performance measures are not interchangeable. The aforementioned discrepancies are intriguing and invite a broader discussion on the applicability of 5-CST and handgrip strength in different settings, especially as consensus discussions on an international definition of sarcopenia proceed [7].

There may be several explanations as to why we find very little agreement between handgrip strength and 5-CST status, independent relationships for each test with the primary outcome, and some evidence of a stronger relationship between 5-CST and mortality. Handgrip strength is a direct measure of muscle strength, and while it has previously been shown to associate with all-cause mortality in the Tromsø Study and elsewhere [1, 29, 30], it is possible that the relationship between 5-CST and mortality is further influenced by several other vital physical capacities that 5-CST performance depend on [13]. Maximal leg strength and power are likely the largest contributors to 5-CST performance, supporting the EWGSOP2 decision to recognise the 5-CST as a clinically available proxy marker and indirect measure of leg strength [6, 12, 13, 31]. It should be noted, however, that studies comparing direct measures of upper- and lower-body muscle strength report inconclusive findings with regard to any differences in premature death risk [4, 30]. Our observations of independent associations could relate to anthropometric differences, as people with poor 5-CST performance typically express more obesity-related characteristics such as increased weight, larger waist circumference and higher %body fat compared with those with weak handgrip strength [15]. The association between 5-CST performance and mortality could be partially driven by participants with a sarcopenic obesity phenotype, which has shown stronger associations with mortality and mobility impairment compared with sarcopenia alone [32, 33]. However, our findings were not attenuated by adjustment for BMI. Regarding our sensitivity analyses, we can only speculate as to why associations between 5-CST performance and mortality appeared more robust to exclusion of early deaths compared with handgrip strength. Reduced handgrip strength could to a larger extent convey pre-existing multimorbidity while reduced 5-CST performance may indicate mobility disability that occurs earlier on the causal pathway leading to premature death [34, 35]. Interestingly, sensitivity analyses also revealed stronger associations between the EWGSOP2 probable sarcopenia cutoff for handgrip strength and mortality, compared with the cutoff from the 5-CST. Although we argue that comparisons between these cutoff points are more difficult to interpret because they were derived in different study samples, with different methods, and only the handgrip strength cutoff included separate values for men and women [6].

A key strength of the present study is the use of *T*-scores that maximises the value of data on adults at different life stages. This also enables standardised comparisons between handgrip strength and 5-CST status and adopts an approach similar to past developments of osteoporosis and sarcopenia definitions [6, 22]. While recent studies have provided normative data for the 5-CST [36, 37], this is the first study to report 5-CST *T*-scores, which can potentially help inform ongoing international sarcopenia definition discussions with cutoffs validated from hard endpoint data as requested by the EWGSOP2 [6]. *T*-scores have previously been reported for the 30-s CST [38], which bears resemblance to the 5-CST but might to a larger extent reflect endurance capacity rather than muscle strength [26]. It is, however, acknowledged that the 30-s CST may be adopted in a wider range of populations because of lower floor and higher ceiling effects, and because there is no requirement of a set number of completed repetitions for the test to be valid [39]. When generating the *T*-scores, we used the youngest age group (40–44 years) in the sample as the reference group, and these might not be considered as “young adults” in comparison with other studies reporting *T*-scores for osteoporosis and sarcopenia definitions [6, 22, 38]. However, Landi and colleagues recently investigated muscle strength measures in a broader age spectrum and reported that handgrip strength and 5-CST performance remained stable up to 40–44 years with an apparent decline thereafter, providing support to our use of this age group as a reference [36]. Interestingly, the present study’s cutoffs for very low handgrip strength (*T*-score − 2.5; men 32.3 kg; women 19.3 kg) are closely aligned with the less conservative cutoff (*T*-score − 2.0; men 32 kg; women 19 kg) proposed by Dodds and colleagues, and used by the EWGSOP2, where several UK cohorts were pooled [5]. Similar comparisons and conclusions were also reported from a Danish cohort [38]. This might be indicative of the Tromsø7 study sample being generally healthier than the pooled UK study samples, as supported by findings that mean handgrip strength is higher in more recently born Tromsø Study participants [40]. To this end, our findings would benefit from being validated in other study populations.

The present study has some limitations. First, the maximum follow-up time of 5.5 years was relatively short and included relatively few deaths (3%) whereby we had limited statistical power especially for analyses of combined effects. It would thus have been valuable to investigate the similarity and strength of the associations over a longer study period to calculate both 5- and 10-year mortality rates and investigate cause-specific mortality [29]. It is also possible the relatively low death rate led to the study being underpowered for sex-stratified analyses, as indicated by the non-significant interaction terms despite some evidence (assessed qualitatively) that associations may differ by sex. Second, while mortality is an important clinical endpoint which due to its ascertainment via linkage to the national death registry is not subject to reporting bias, it is only one of several potentially important clinical endpoints relevant when considering the role of HGS and 5-CST in relation to probable sarcopenia diagnosis. Future research examining additional endpoints including hospitalisation, falls, fractures and mobility disability may therefore be beneficial. Third, the study involved independent community-dwelling participants and the findings may thus not be generalisable to institutionalised or care-dependent older adults. Additionally, we were not able to incorporate responses to the SARC-F questionnaire as per EWGSOP2 guidelines [6], as this instrument has not been used in the Tromsø Study. The SARC-F is intended as a first line screening in the sarcopenia case-finding algorithm and its absence may have led to a lower prevalence of probable sarcopenia in the study population. We also cannot rule out selection bias from our analyses as 35% of participants invited to basic examinations in Tromsø7 declined participation and this potential selection would have followed the randomised sub-sample invited to extended examinations. Recently published Tromsø7 data indicate that non-attendees were more likely to live alone, have lower socioeconomic status, and belong to the youngest and oldest age groups [41]. Finally, even though we included relevant lifestyle and disease covariates, our analyses might still have been subject to residual confounding; the contemporaneous nature of self-reported parameters such as smoking, disease status, and leisure time physical activity may not reflect all relevant aspects of lifetime exposure.

## Conclusions

*T*-score categories of handgrip strength and 5-CST performance showed very little agreement and their associations with mortality were independent of each other. Our findings indicate that these tests cannot be used interchangeably when case finding probable sarcopenia, as they potentially identify different people at risk. As discussions on an international consensus for sarcopenia definitions proceed, assessment of both handgrip strength and chair stand performance to identify probable sarcopenia should be recommended rather than these tests being viewed as interchangeable.

### Supplementary Information

**Additional file 1: Tables S1-S3. Table S1 – **Complete case analysis of the study sample in Cox proportional hazard regression models. The Tromsø Study 2015-2020. **Table S2 – **Sensitivity analysis of Cox proportional hazard regression models excluding deaths occurring during the first two years of follow-up (*N* = 7772). The Tromsø Study 2015-2020. **Table S3 – **Sensitivity analysis of Cox proportional hazard regression models investigating associations between EWGSOP2 cutoff points and mortality (*N* = 7838). The Tromsø Study 2015-2020.

## Data Availability

The dataset supporting the article findings is available through application directed to the Tromsø Study, by following the steps presented on its online page: https://uit.no/research/tromsostudy

## Abbreviations

5-CST: Five-repetition chair stand test
BMI: Body mass index
CI: Confidence interval
EWGSOP2: European Working Group on Sarcopenia in Older People
HR: Hazard ratio
SD: Standard deviation
