# Supplementary material for: Comparing associations of handgrip strength and chair stand performance with all-cause mortality—implications for defining probable sarcopenia: the Tromsø Study 2015–2020
Source: BMC Med. 2023 Nov 20;21:451. doi: 10.1186/s12916-023-03172-3 (PMC10659040; doi:10.1186/s12916-023-03172-3)
Supplement: Supplementary file 1 — Additional file 1: Tables S1-S3. Table S1 – Complete case analysis of the study sample in Cox proportional hazard regression models. The Tromsø Study 2015-2020. Table S2 – Sensitivity analysis of Cox proportional hazard regression models excluding deaths occurring during the first two years of follow-up (N = 7772). The Tromsø Study 2015-2020. Table S3 – Sensitivity analysis of Cox proportional hazard regression models investigating associations between EWGSOP2 cutoff points and mortality (N = 7838). The Tromsø Study 2015-2020. [file 12916_2023_3172_MOESM1_ESM.docx]

**Additional file 1**

**Article title:** Comparing associations of handgrip strength and chair stand performance with all-cause mortality - implications for defining probable sarcopenia: The Tromsø Study 2015-2020

**Journal name:** BMC Medicine

**Author names:** Jonas Johansson, Sameline Grimsgaard, Bjørn Heine Strand, Avan A. Sayer, Rachel Cooper

**Affiliation and e-mail address of corresponding author:** Jonas Johansson (corresponding author). E-mail: jonas.johansson@uit.no. Affiliation: Department of Community Medicine, UiT The Arctic University of Norway, Tromsø, Norway

**Table S1** Complete case analysis of the study sample in Cox proportional hazard regression models. The Tromsø Study 2015-2020

| **Variable** | **Model 1 (n=7838)** | | **Model 2 (n=6949)** | | **Model 3 (n=6949)** | |
| --- | --- | --- | --- | --- | --- | --- |
|  | **HR** | **95% CI** | **HR** | **95% CI** | **HR** | **95% CI** |
| Handgrip strength |  |  |  |  |  |  |
| Not low | 1.00 | Ref. | 1.00 | Ref. | 1.00 | Ref. |
| Low | 1.30 | 0.94, 1.79 | 1.25 | 0.86, 1.83 | 1.15 | 0.79, 1.69 |
| Very low | 2.23 | 1.44, 3.47 | 2.41 | 1.40, 4.17 | 1.82 | 1.02, 3.23 |
|  |  |  |  |  |  |  |
| 5-CST performance |  |  |  |  |  |  |
| Not low | 1.00 | Ref. | 1.00 | Ref. | 1.00 | Ref. |
| Low | 2.09 | 1.55, 2.81 | 1.74 | 1.22, 2.49 | 1.68 | 1.18, 2.40 |
| Very low | 3.51 | 2.41, 5.11 | 2.83 | 1.75, 4.58 | 2.42 | 1.46, 4.02 |

HR, hazard ratio; CI, confidence interval; 5-CST, 5-repetition chair stand test

Model 1: adjusted for age (as timescale) and sex

Model 2: adjusted for model 1 + height, BMI, leisure time physical activity, education, smoking status, cardiovascular disease, rheumatoid arthritis, respiratory disease

Model 3: adjusted for model 1 and model 2 + handgrip strength status or 5-CST status

**Table S2** Sensitivity analysis of Cox proportional hazard regression models excluding deaths occurring during the first two years of follow-up (N = 7772). The Tromsø Study 2015-2020

| **Variable** | ***N*** | **Deaths** | **MR^a^** | **Model 1** | | **Model 2^b^** | | **Model 3^b^** | |
| --- | --- | --- | --- | --- | --- | --- | --- | --- | --- |
|  |  |  |  | **HR** | **95% CI** | **HR** | **95% CI** | **HR** | **95% CI** |
| Handgrip strength |  |  |  |  |  |  |  |  |  |
| Not low | 4177 | 48 | 2.43 | 1.00 | Ref. | 1.00 | Ref. | 1.00 | Ref. |
| Low | 3232 | 95 | 6.23 | 1.08 | 0.74, 1.57 | 1.11 | 0.76, 1.64 | 1.01 | 0.69, 1.50 |
| Very low | 363 | 24 | 14.23 | 1.46 | 0.85, 2.49 | 1.54 | 0.87, 2.73 | 1.20 | 0.66, 2.18 |
|  |  |  |  |  |  |  |  |  |  |
| 5-CST performance |  |  |  |  |  |  |  |  |  |
| Not low | 5569 | 65 | 2.48 | 1.00 | Ref. | 1.00 | Ref. | 1.00 | Ref. |
| Low | 1815 | 70 | 8.12 | 1.93 | 1.36, 2.74 | 1.77 | 1.23, 2.53 | 1.76 | 1.22, 2.52 |
| Very low | 388 | 32 | 17.79 | 2.79 | 1.78, 4.38 | 2.35 | 1.45, 3.80 | 2.24 | 1.35, 3.71 |

MR, mortality rate; HR, hazard ratio; CI, confidence interval; 5-CST, 5-repetition chair stand test

^a^ Mortality rate per 1000 person-years

^b^ Estimates are from models run across 30 imputed datasets combined using Rubin’s rules

Model 1: adjusted for age (as timescale) and sex

Model 2: adjusted for model 1 + height, BMI, leisure time physical activity, education, smoking status, cardiovascular disease, rheumatoid arthritis, respiratory disease

Model 3: adjusted for model 1 and model 2 + handgrip strength status or 5-CST status

**Table S3** Sensitivity analysis of Cox proportional hazard regression models investigating associations between EWGSOP2 cutoff points and mortality (N = 7838). The Tromsø Study 2015-2020

| **Variable** | ***N*** | **Deaths** | **MR^a^** | **Model 1** | | **Model 2^b^** | | **Model 3^b^** | |
| --- | --- | --- | --- | --- | --- | --- | --- | --- | --- |
|  |  |  |  | **HR** | **95% CI** | **HR** | **95% CI** | **HR** | **95% CI** |
| Handgrip strength |  |  |  |  |  |  |  |  |  |
| Not sarcopenia | 7727 | 213 | 5.87 | 1.00 | Ref. | 1.00 | Ref. | 1.00 | Ref. |
| Probable sarcopenia | 111 | 20 | 41.76 | 3.24 | 2.01, 5.20 | 3.54 | 2.15, 5.84 | 3.17 | 1.91, 5.28 |
|  |  |  |  |  |  |  |  |  |  |
| 5-CST |  |  |  |  |  |  |  |  |  |
| Not sarcopenia | 7358 | 181 | 5.24 | 1.00 | Ref. | 1.00 | Ref. | 1.00 | Ref. |
| Probable sarcopenia^c^ | 480 | 52 | 23.88 | 2.37 | 1.70, 3.29 | 2.01 | 1.42, 2.84 | 1.85 | 1.31, 2.63 |
|  |  |  |  |  |  |  |  |  |  |

MR, mortality rate; HR, hazard ratio; CI, confidence interval; 5-CST, 5-repetition chair stand test

^a^ Mortality rate per 1000 person-years

^b^ Estimates are from models run across 30 imputed datasets combined using Rubin’s rules

^c^ Participants unable to complete the 5-CST were added to this category

Model 1: adjusted for age (as timescale) and sex

Model 2: adjusted for model 1 + height, BMI, leisure time physical activity, education, smoking status, cardiovascular disease, rheumatoid arthritis, respiratory disease

Model 3: adjusted for model 1 and model 2 + probable sarcopenia status by handgrip strength or 5-CST
